# Medial meniscus extrusion during gait identifies altered knee mechanical loading in early osteoarthritis of the knee

**DOI:** 10.1002/jeo2.70910

**Published:** 2026-09-15

**Authors:** Yosuke Ishii, Kyohei Nakata, Akinori Nekomoto, Atsuo Nakamae, Masakazu Ishikawa, Yuko Nakashima, Shigeru Sato, Naoto Fujita, Nobuo Adachi

**Affiliations:** ^1^ Department of Bio‐Environmental Adaptation Sciences, Graduate School of Biomedical and Health Sciences Hiroshima University Hiroshima Japan; ^2^ Department of Orthopedic Surgery, Graduate School of Biomedical and Health Sciences Hiroshima University Hiroshima Japan; ^3^ Department of Orthopaedic Surgery, Faculty of Medicine Kagawa University Kagawa Japan; ^4^ Department of Translational Education and Research Support Center for Medical Sciences Hiroshima University Hiroshima Japan; ^5^ Department of Sports Medical Center Hiroshima University Hospital Hiroshima Japan

**Keywords:** early‐stage, knee adduction moment, knee osteoarthritis, medial meniscus extrusion, walking

## Abstract

**Purpose:**

This study aimed to develop a modified knee adduction moment (KAM) measure as a composite measure of mechanical loading, incorporating medial meniscal extrusion (MME), and investigate whether it could distinguish patients with the biomechanical pathology of early knee osteoarthritis (OA) from healthy individuals.

**Methods:**

This study included 23 patients with early knee OA (Kellgren–Lawrence grade 1–2), 14 healthy young (younger group) and 13 healthy elderly individuals (older group). In patients with knee OA, knee alignment was evaluated using hip‐knee‐ankle angle. For investigating the gait‐related mechanical loading, the KAM, including the impulse value, was measured during walking using a motion analysis system synchronised with an ultrasound. MME waveforms were generated from continuous MME values obtained using ultrasound in video mode, and the difference between the minimum and maximum values of the MME behaviour was shown as the ΔMME. Furthermore, the modified KAM was calculated by multiplying the KAM impulse by the ΔMME value.

**Results:**

No significant differences were observed in the KAM impulse among all groups. ΔMME and modified KAM were significantly higher in the knee OA group than in the younger and older groups. In the knee OA group, a significant correlation was observed only between the modified KAM and the hip‐knee‐ankle angle, whereas no significant correlation was found for the KAM or ΔMME alone.

**Conclusions:**

The modified KAM identified biomechanical features associated with mechanical loading stress in the medial compartment in patients with early‐stage knee OA.

**Level of Evidence:**

Level IV.

## Graphical abstract

The illustration was created from an original schematic prepared by the authors and refined using ChatGPT (OpenAI, 2026). The authors reviewed and edited the final illustration for scientific accuracy.

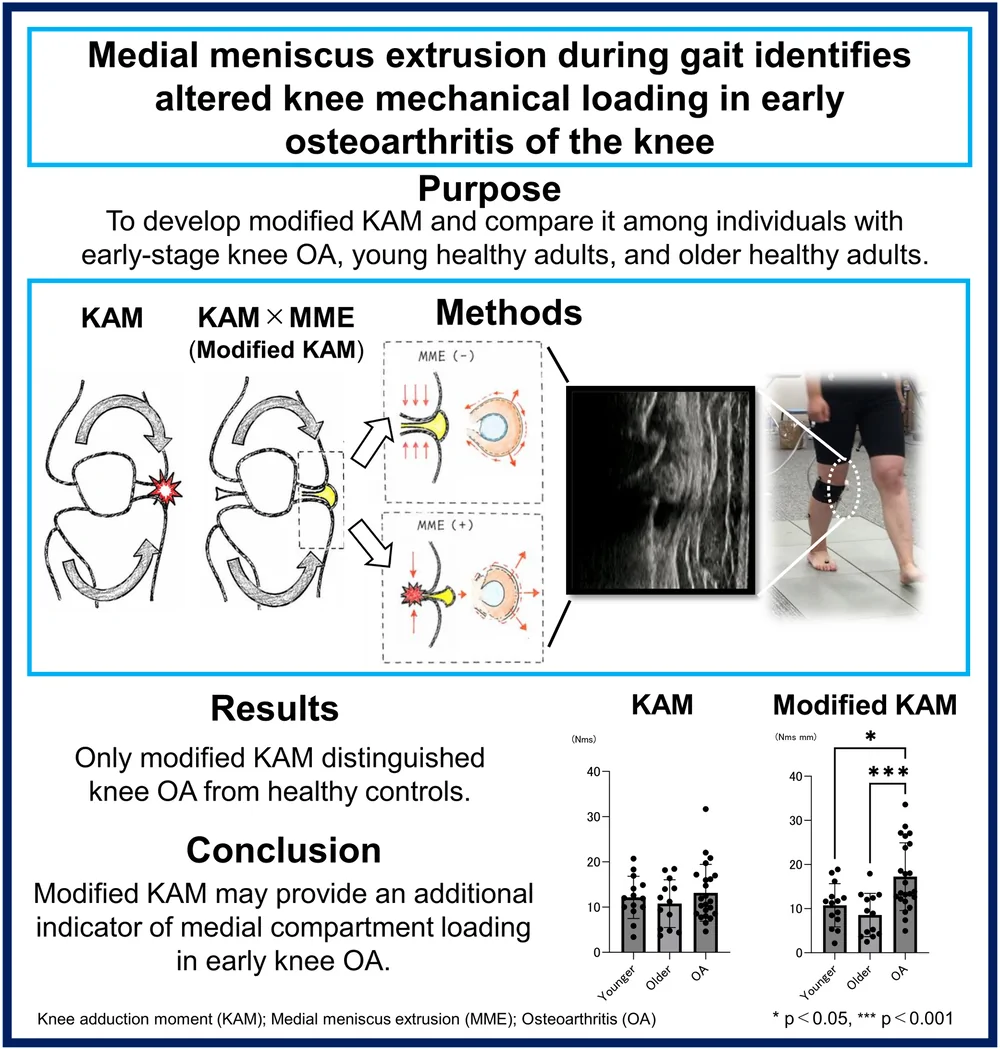

**Abbreviations**
%MA: percentage of the mechanical axis
HKAA: hip‐knee‐ankle angle
JLCA: joint line convergence angle
K/L: Kellgren–Lawrence
KAM: knee adduction moment
MME: medial meniscus extrusion
MTPA: medial proximal tibial angle
OA: knee osteoarthritis
ΔMME: dynamic behaviour of medial meniscus extrusion

## INTRODUCTION

Knee osteoarthritis (OA) is a degenerative joint disease that progresses through the interaction of multiple factors, including inflammation, static alignment and mechanical loading [12]. Among these factors, cumulative abnormal mechanical stress is considered the major driver of disease progression because it induces structural alterations within the medial compartment over time [1, 8]. Therefore, identifying pathological mechanical loading during the early stages of OA may be important for preventive and disease‐modifying interventions.

The knee adduction moment (KAM) is one of the most widely used biomechanical indicators of medial compartment loading during walking [36]. Greater KAM is associated with knee OA progression and has been used as a surrogate measure of knee joint loading [26, 31, 39]. However, evidence regarding the ability of KAM to detect early‐stage OA is inconsistent [20, 30]. Moreover, KAM primarily reflects external loading and does not account for the functional status of intraarticular tissues that modulate load transmission within the joint.

In the knee joint, the meniscus plays a critical role in load distribution and shock absorption through its hoop function [37]. Degeneration and tearing of the medial meniscus can impair meniscal function and lead to medial meniscal extrusion (MME) [6, 9]. Abnormal MME has been observed even at the earlier stages of the knee OA [10], suggesting that meniscal dysfunction may substantially modify structural disease progression.

Although both KAM and MME have been investigated in relation to knee joint loading, the association between them appears to be moderate and dependent on the population studied, which could represent different aspects of knee joint mechanics [17, 41]. Importantly, the MME alters under different mechanical loading conditions [34, 40]. An increase in MME from an unloaded condition to a loaded condition, such as from the supine position to standing, or during walking, has been described as meniscal behaviour and may reflect the dynamic response of the meniscus to mechanical stress [17, 21]. Notably, in individual patients, meniscal behaviour varies with meniscus quality, such as degeneration and tear [2]. Previous studies have demonstrated that dynamic meniscal behaviour is associated with cartilage loss, OA progression and symptoms rather than the absolute MME measured under static conditions, indicating that it may provide functional information beyond static morphological assessment of the meniscal hoop function [5, 32, 33]. Therefore, incorporating meniscal behaviour into KAM may complement and enhance the characterisation of pathological mechanical loading conditions, particularly at the early stages of OA.

Static varus alignment is a well‐established radiographic marker associated with knee OA progression and cumulative medial compartment loading [14, 42]. Therefore, the relationship between a modified KAM, a composite measure of biomechanical loading, and varus alignment may provide insights into its validity as an indicator of OA‐related mechanopathology. This study aimed to develop a modified KAM as a composite biomechanical measure and compare its values across individuals with early‐stage knee OA, young healthy adults and older healthy adults. The secondary aim was to investigate the associations of the modified KAM, KAM impulse and MME behaviour with radiographic alignment in the OA group and to compare the strengths of these associations to assess the potential biomechanical relevance of the modified KAM. The hypothesis was that KAM modified by MME behaviour would distinguish early‐stage knee OA more effectively than the conventional KAM and would be associated with radiographic varus alignment.

## MATERIALS AND METHODS

### Participants

The patients were diagnosed with unilateral or bilateral tibiofemoral knee OA based on radiography and were included in this study. Kellgren–Lawrence (K/L) scores were used to assess OA severity, and stages 1 and 2 were identified as early knee OA (Table 1). In patients with bilateral tibiofemoral knee OA, the index knee was defined as the knee with more symptoms and severity than the other. Individuals requiring any support during walking, a history of surgical treatment and trauma on the index knee, neurological disorder, lack of magnetic resonance image (MRI) data and moderate or severe stage of knee OA (K/L ≧ 3) were excluded from this study. Particularly, patients with acute medial meniscus posterior root tear (MMPRT) were also excluded because of the direct effect on meniscal behaviour through complete destruction of the hoop structure. In contrast, young and older healthy individuals were enrolled into the younger and older control groups; consequently, this study included three groups (Table 1).

**Table 1:** Demographic data.

Variable	Younger (*n* = 14)	Older (*n* = 13)	OA (*n* = 23)	*p*‐value
Age (year)	22.07 ± 1.10	61.54 ± 8.03 ^a^	61.04 ± 11.73 ^a^	<0.001
Height (m)	1.65 ± 0.04	1.66 ± 0.09	1.63 ± 0.08	0.4218
Weight (kg)	57.95 ± 5.75	63.64 ± 9.48	64.88 ± 12.05	0.1388
BMI (kg/m^2^)	21.34 ± 1.70	22.96 ± 2.37	24.37 ± 3.36 ^a,b^	0.01012
Sex (male, female)	5, 9	7, 6	13, 10	—
K/L (I, II)	—	—	2,21	—
HKAA (deg)	—	—	−4.7 ± 3.1	—
%MA (%)	—	—	31.0 ± 11.5	—
MTPA (deg)	—	—	84.0 ± 1.4	—
JLCA (deg)	—	—	1.7 ± 0.8	—

*Note*: Values are presented as mean ± standard deviation. a, b, significantly higher than the younger or the older group (*p* < 0.05). *p*‐values show statistical differences among groups using one‐way analysis of variance or Kruskal–Wallis test.

Abbreviations: %MA, percentage of mechanical axis; BMI, body mass index; HKAA, hip–knee–ankle angle; JLCA, joint line convergence angle; K/L, Kellgren–Lawrence; MPTA, medial proximal tibial angle; n, sample size; OA, osteoarthritis.

Our institutional review board approved this study, and all participants provided written informed consent.

### Radiographical assessment

Patients with knee OA underwent radiographic assessment of lower‐limb alignment. On full‐length weight‐bearing radiographs, a single examiner assessed coronal lower‐limb alignment using the hip–knee–ankle angle (HKAA), percentage of mechanical axis (%MA), medial proximal tibial angle (MPTA) and joint line convergence angle (JLCA), according to previously described methods [15, 25]. The HKAA was defined as the angle formed by the mechanical axes of the femur and the tibia. The %MA was defined as the percentage position at which the mechanical axis of the lower limb crossed the proximal tibial joint line. The MPTA was defined as the medial angle between the tibial mechanical axis and proximal tibial joint line, whereas the JLCA was defined as the angle formed by the distal femoral and proximal tibial joint lines. Negative HKAA values and lower %MA and MPTA values indicated greater varus alignment. The reliability of these radiographic measurements was previously evaluated in a pilot study [16]. In that study, the measurements were independently performed by two orthopaedic surgeons, each with more than 10 years of clinical experience, and interrater reliability was assessed using ICC (2,1). The ICC (2,1) values were 0.99 (95% confidence interval [CI]: 0.96–1.00) for HKAA, 0.91 (95% CI: 0.76–0.97) for %MA, 0.74 (95% CI: 0.39–0.91) for MPTA, and 0.74 (95% CI: 0.39–0.91) for JLCA [16].

### Assessment of the meniscus quality

To assess the presence and location of meniscal tears, MRI scans obtained as part of routine clinical care within 3 months after the initial consultation were reviewed for patients with knee OA. The MRI images were interpreted by an orthopaedic surgeon specialising in knee disorders and having more than 5 years of clinical experience, who determined the presence and location of meniscal tears. The type of meniscal tear was evaluated based on any one of the criteria, and classified as horizontal, vertical, longitudinal, complex and degenerative. The tear patterns were categorised according to the orientation and morphological characteristics of the abnormal intrameniscal signals. Horizontal tears were characterised by a linear signal extending along a plane approximately parallel to the tibial plateau. Vertical and longitudinal tears were classified according to their orientation and course relative to the circumferential axis of the meniscus. Complex tears were defined by the presence of more than one tear pattern, whereas degenerative tears were characterised by irregular and heterogeneous signal abnormalities with the disruption of normal meniscal morphology. Especially, the examiner carefully confirmed the presence of the MMPRT. These abnormal features were identified based on the giraffe neck and ghost sign, according to previous reports [11, 28].

### Gait analyses

Kinetic and kinematic data were obtained using three‐dimensional motion analysis system consisted of 16 cameras (Vicon Motion Systems) and eight force plates (AMTI) and equipped with the sampling rate of 100–1000 Hz. Based on the plug‐in‐gait lower‐body marker set, participants were attached with 16 reflective markers on the anatomical landmarks on the lower body. Trials of walking at a comfortable speed were performed twice for each patient. Force plates were used to detect the gait events of the participants, including initial contact and toe‐off, using the threshold force of 10 N. Knee angles and moments in the stance phase were calculated using Nexus 1.8.5 software. These values were standardised to 101 points for each task. The outcomes were coronal knee motion and moments with knee varus angles and KAMs. Peak values and impulses were recorded, and representative values were calculated based on the average of the trials. Varus thrust was defined as the difference between the minimum and maximum varus angles until 20% stance‐phase. Additionally, the gait speed was calculated at different times on the heel marker between the initial contacts. All analyses were performed using MATLAB R2020a (MathWorks).

### The evaluation of medial meniscus extrusion

**Figure 1:**
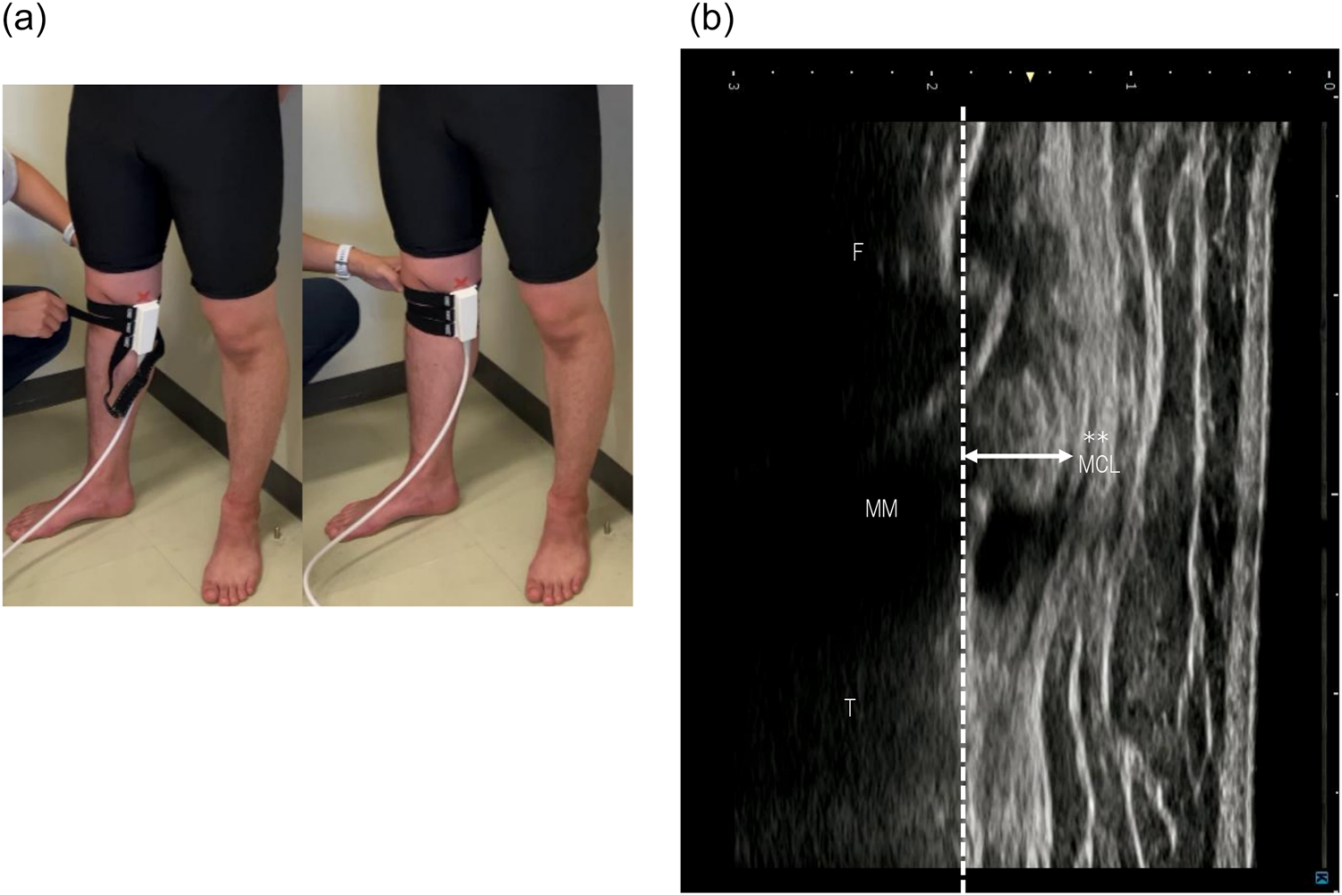
Representative ultrasound images with a prototype transducer. (a) Transducer attachment, and (b) the representative image of medial meniscus extrusion. The white line and double arrows represent the cortical line of the tibial plateau and distance of the extruded medial meniscus, respectively. F, femur; MCL, medial collateral ligament; MM, medial meniscus; T, tibia.

The medial meniscus was dynamically evaluated during gait using ultrasonography. An ultrasound device (SNiBLE, KONICA MINOLTA) equipped with a specialised prototype 3–11 MHz linear transducer was used to capture real‐time images of the medial knee compartment. The transducer was aligned longitudinally along the medial joint line and secured in a position that allowed for continuous visualisation of the medial meniscus during walking (Figure 1a).

To standardise image acquisition across participants, the superior margin of the medial femoral condyle and the vertical line were first identified and used as a reference landmark. The transducer position was subsequently optimised to obtain a distinct image of the medial meniscus and adjacent medial collateral ligament before data collection commenced and fixed by a special holder with a flexible band (Figure 1a). Ultrasound recordings were obtained at 30 frames per second while participants walked at a self‐selected speed. A synchronisation event was generated by placing a detectable signal in both the ultrasound and motion capture systems. Kinematic and kinetic data were collected simultaneously using a motion analysis system and force plates.

**Figure 2:**
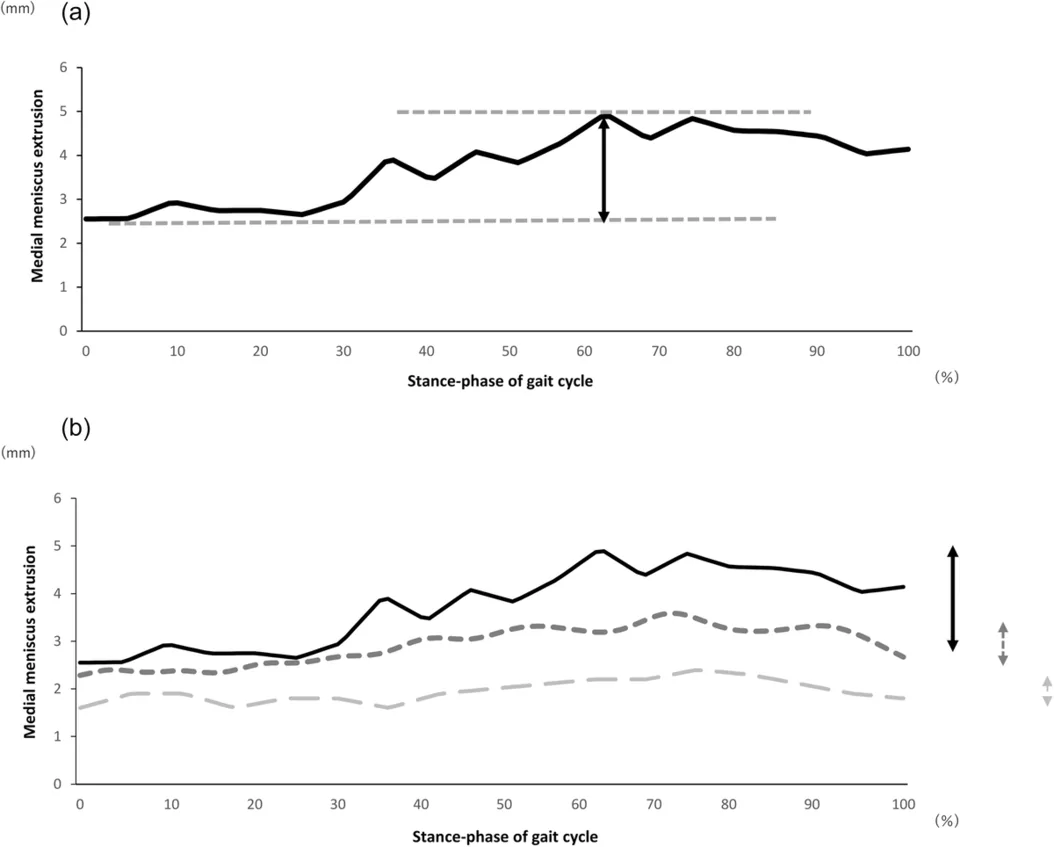
Waveform of medial meniscus extrusion during walking. (a) The representative waveforms of meniscal extrusion during walking, and (b) individual waveforms in each group. The dotted lines and double black arrows show the maximum and minimum values of meniscal extrusion and the behaviour of meniscal extrusion during walking (a). The black and dotted lines with black and grey lines represent the waveform of meniscal extrusion in patients with knee osteoarthritis and older and younger healthy individuals, respectively. Double arrows indicate the behaviour of meniscal extrusion in each individual.

For image analysis, MME was determined as the perpendicular distance from the cortical line of the medial tibial plateau to the outermost edge of the medial meniscus [17] (Figure 1b). Multiple ultrasound frames were extracted from each walking trial, and MME was quantified in every frame. To identify MME during gait, the ΔMME was calculated as the increase in the MME values observed throughout the stance‐phase, and is defined as the difference between the minimum at initial contact and the maximum during a trial (Figure 2). The minimum‐to‐maximum difference was selected as a simple and reproducible measure of meniscal excursion. This approach accounted for the influence of knee flexion on ultrasound‐based MME measurement: flexion‐induced posterior translation of the medial meniscus may alter the portion of the meniscus visualised in the fixed imaging plane and thereby affect the measured MME. Because the minimum MME value at initial contact and the maximum MME value later in the stance phase were measured while the knee was relatively extended, the difference between these values was considered to be a feasible measure of increased extrusion of the medial meniscal body while minimising the influence of posterior meniscal translation associated with knee flexion. Analysis was performed using the Kinovea software (version 0.8.15, Kinovea Open Source Project). This measurement procedure has been previously described and shown to have good intrarater and interrater reliability for MME measurements [17]. In addition, previous studies using the same measurement method have shown that ΔMME was responsive to changes in mechanical loading following conservative and surgical interventions [18, 19], thereby providing indirect support for its validity as a biomechanically relevant measure during gait.

### The establishment of the modified KAM

**Figure 3:**
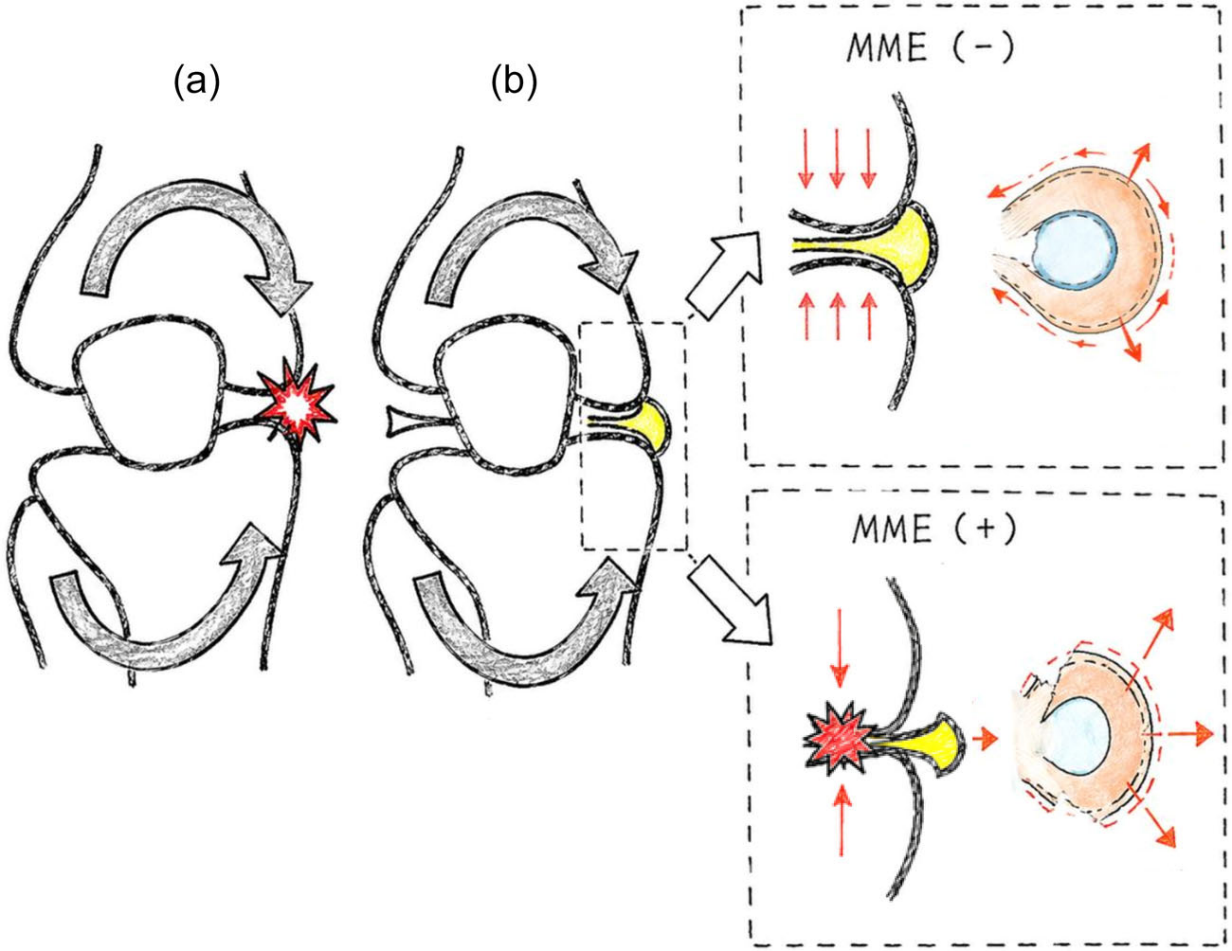
Schema of modified knee adduction moment (KAM). Range of KAM (a) and modified meniscus information (modified KAM) (b). The right upper and lower images show normal and abnormal hoop functions of the medial meniscus. The modified KAM was calculated as the product of the KAM impulse and ΔMME, integrating the cumulative external knee loading during the stance phase with the magnitude of the dynamic meniscal extrusion. MME, medial meniscal extrusion; ΔMME, the difference in medial meniscus extrusion between the minimum and maximum during the stance phase. The illustration was created from an original schematic prepared by the authors and refined using ChatGPT (OpenAI 2026). The authors reviewed and edited the final illustrations for scientific accuracy.

To establish a composite biomechanical index, the KAM impulse was multiplied by ΔMME. The KAM impulse represents the cumulative external mechanical loading of the medial knee compartment during the stance phase, whereas ΔMME represents the dynamic response of the medial meniscus to loading and may reflect its load‐distribution function. This index assumes that the mechanical burden on the medial knee compartment depends not only on the magnitude and duration of external loading but also on the functional response of the meniscus to that loading. Therefore, the KAM impulse and ΔMME were combined multiplicatively to integrate cumulative external knee loading with the dynamic meniscal response. This approach emphasises the mechanical conditions under which high cumulative external loading and greater dynamic meniscal extrusion coexist. The KAM impulse value was thereby modified according to the individual meniscal response to loading during the stance phase of the gait cycle (Figure 3). The value of the modified KAM was compared with conventional KAM parameters and ΔMME.

### Statistical analysis

The normal distribution of all variables was assessed using the Shapiro–Wilk test. The primary analysis was a comparison of the modified KAM scores among the three groups. The exploratory analyses included between‐group comparisons of demographic characteristics, KAM impulses, MME and ΔMME. These variables were compared among the groups using one‐way analysis of variance for normally distributed data or the Kruskal–Wallis test for nonnormally distributed data. Effect sizes were interpreted using conventional benchmarks of 0.01, 0.06 and 0.14 for small, medium, and large *ω* ^2^ effects, respectively [23]. Pairwise comparisons were performed using Tukey's or the Dunn–Bonferroni test, as appropriate. In addition, as secondary analyses, the relationships between radiographic knee alignment parameters and biomechanical measures, including the modified KAM, KAM impulse and ΔMME, within the OA group were examined using Pearson's or Spearman's correlation coefficients, as appropriate. All statistical analyses were performed using R (version 4.4.2). Statistical significance was defined as *p* < 0.05.

## RESULTS

### Demographic data and characteristics of knee OA

This study finally sampled 23 patients with knee OA (mean age, 61.0 ± 11.7 years; 13 males and 10 females), 14 young (mean age, 22.0 ± 1.1 years; 5 males and 9 females) and 13 older (mean age, 61.5 ± 8.0 years; 7 males and 6 females) healthy individuals. The demographic data are shown in Table 1. Furthermore, body mass index (BMI) was significantly higher in the OA group than in the younger and older healthy groups (Table 1).

Most participants in the OA group were classified as K/L grade 2 and exhibited varus knee alignment with a medially shifted mechanical axis (HKAA: −4.7 ± 3.1 degrees; %MA: 31.0 ± 11.5%) (Table 1). MRI demonstrated an abnormal signal intensity within the medial meniscus in all patients with knee OA. Degenerative meniscal lesions were the most frequently observed abnormalities among other meniscal pathologies (vertical, *n* = 3/23, 13%; horizontal, *n* = 4/23, 17%; oblique, *n* = 1/23, 4%; complex, *n* = 1/23, 4%; degenerative, *n* = 14/23, 61%). Moreover, the most frequent location was the posterior body of the meniscus (body, *n* = 1/23, 4%; posterior body, *n* = 15/23, 65%; posterior horn, *n* = 7/23, 30%).

### Kinematics and kinetics during walking

**Figure 4:**
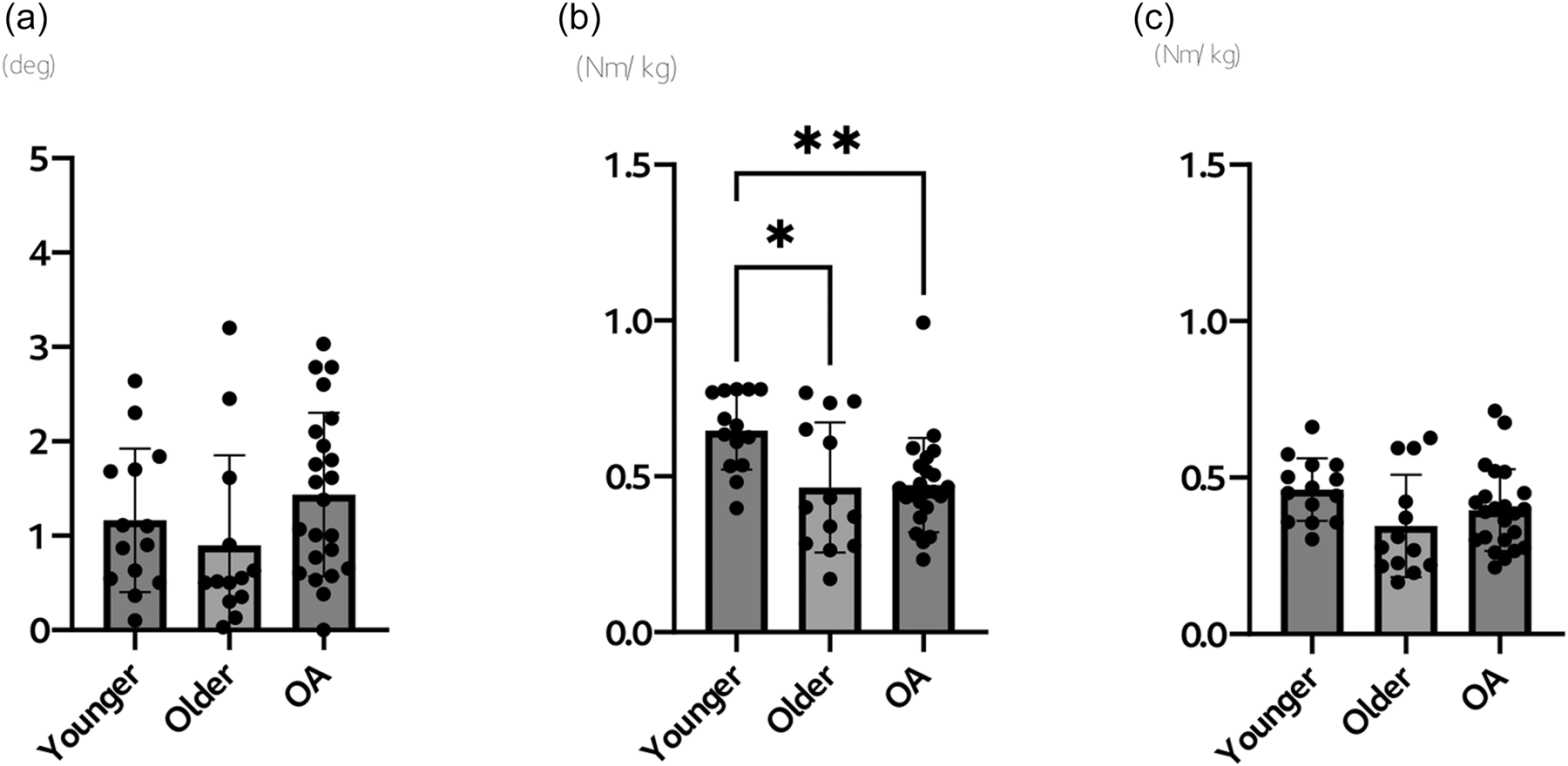
The knee adduction moments. The graphs show the varus thrust (a), and peaks of the knee adduction moment with first (b) and second (c). * and ** represents the significant difference between groups (*p* < 0.05 or *p* < 0.01). OA, osteoarthritis.

The younger group exhibited a significantly higher walking speed and peak first KAM than the OA and older group. However, no significant differences were observed among the groups in varus thrust, vertical ground reaction force or the second peak and KAM impulse (Table 2 and Figure 4).

**Table 2:** Gait parameters.

Variable	Younger	Older	OA	*p*‐value	ES	CI
Varus thrust (deg)	1.16 ± 0.73	0.90 ± 0.91	1.44 ± 0.85	0.07414	0.068	−0.030–0.317
FMP (Nm/kg)	0.54 ± 0.12 ^a,b^	0.46 ± 0.20	0.47 ± 0.15	0.0019	0.224	0.072–0.468
SMP (Nm/kg)	0.46 ± 0.10	0.35 ± 0.16	0.40 ± 0.13	0.04788	0.087	−0.022–0.352
Impulse (Nms)	12.16 ± 4.50	10.78 ± 5.08	13.16 ± 6.14	0.6685	−0.0	‐0.041–0.160
GRF‐z (N)	627.86 ± 73.83	659.43 ± 94.30	649.33 ± 119.58	0.7215	−0.028	−0.040–0.119
gait speed (m/s)	1.12 ± 0.14 ^a,b^	0.92 ± 0.09	0.88 ± 0.15	<0.001	0.350	0.171–0.569

*Note*: Values are presented as mean ± standard deviation. a, b, significantly higher than the older group or the OA group (*p* < 0.05). *p*‐values show statistical differences among groups using one‐way analysis of variance or Kruskal–Wallis test.

Abbreviations: CI, 95% confidence interval; ES, effect size; FMP, first peak of knee adduction moment; GRF‐z, vertical direction of the ground reaction force; Impulse, impulse of knee adduction moment; OA, osteoarthritis; SMP, second peak of knee adduction moment.

### Meniscal extrusion during walking and modified KAM

**Figure 5:**
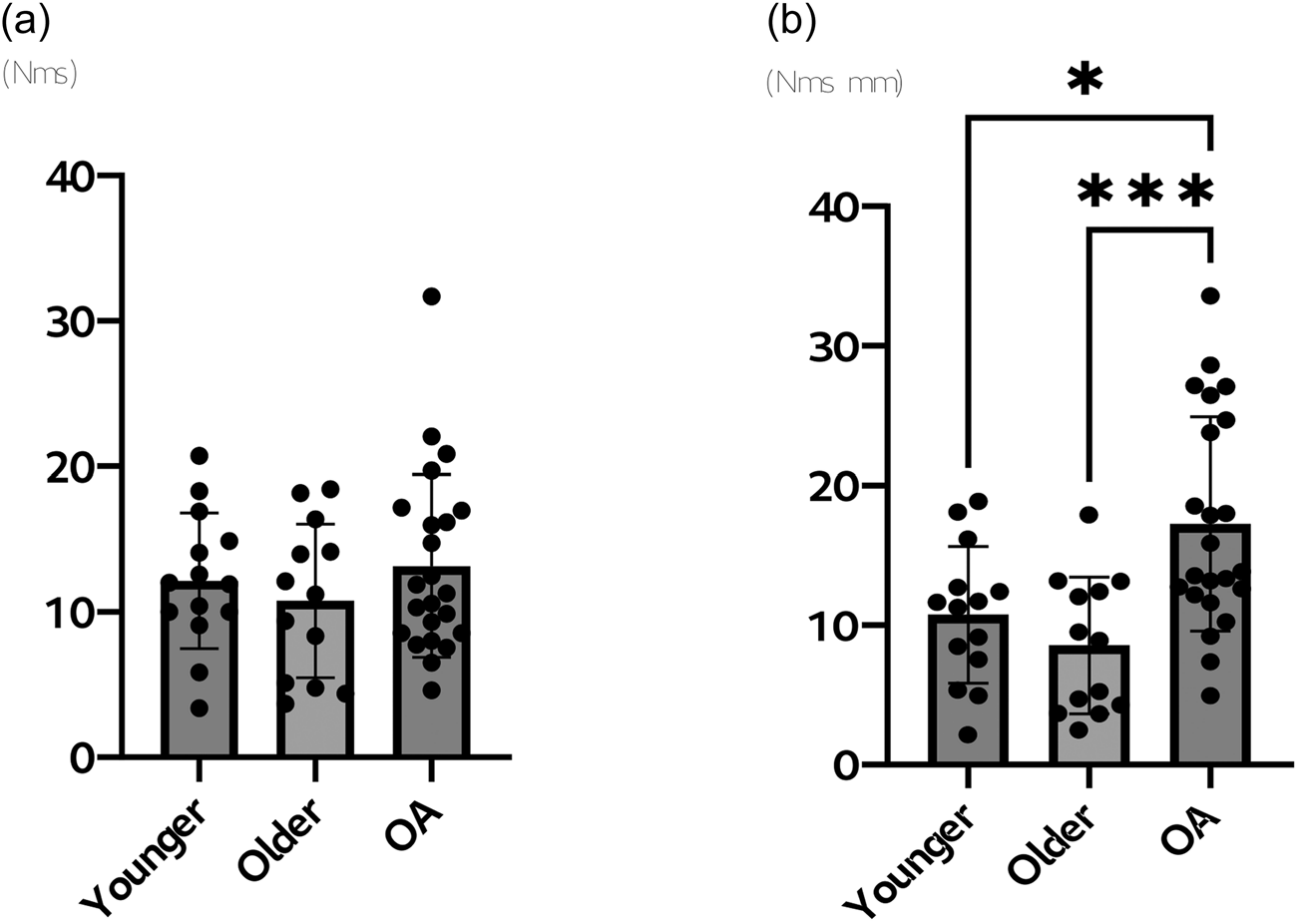
Modified knee adduction moment. The graphs show the impulse of knee adduction moment (a) and the modified knee adduction moment (b). * and *** mean the significant difference between groups (*p* < 0.05 or *p* < 0.001). OA, osteoarthritis.

All MME‐related parameters during walking, including ΔMME, were significantly higher in the OA group than in the young and older healthy groups (Table 3). Furthermore, the modified KAM, calculated as the product of KAM impulse and ΔMME, was significantly higher in the OA group, whereas no significant group difference was observed in KAM impulse (Figure 5).

**Table 3:** Meniscus extrusion and modified knee adduction moment during walking.

Variable	Younger	Older	OA	*p*‐Value	ES	CI
Modified KAM (Nms・mm)	10.75 ± 4.73	8.55 ± 4.70	17.24 ± 7.51 ^a,b^	<0.001	0.247	0.086–0.450
Minimum MME (mm)	1.26 ± 0.89	2.13 ± 0.35	3.12 ± 1.30 ^a,b^	<0.001	0.397	0.161–0.684
Maximum MME (mm)	2.12 ± 1.00	2.98 ± 0.57	4.52 ± 1.58 ^a,b^	<0.001	0.424	0.180–0.702
ΔMME (mm)	0.87 ± 0.19	0.86 ± 0.31	1.39 ± 0.50 ^a,b^	<0.001	0.277	0.082–0.542

*Note*: Values are presented as mean ± standard deviation. a, b: significantly higher than that in the younger or older group (*p* < 0.05). *p*‐values show statistical differences among groups using one‐way analysis of variance or Kruskal–Wallis test.

Abbreviations: CI, 95% confidence interval; ES, effect size; MME, medial meniscus extrusion; Modified KAM, modification between knee adduction moment and ΔMME; OA, osteoarthritis; ΔMME, the difference in medial meniscus extrusion between the minimum and maximum.

The primary outcome of modified KAM revealed a significant group effect (*F* = 9.18, *p* < 0.001). The effect size was large (*ω* ^2^ = 0.247, 95% CI: 0.086–0.450). A post hoc power analysis based on the observed effect and current sample size indicated a statistical power of 0.949, suggesting that the study was adequately powered to detect statistical differences between the groups.

### The correlation with varus alignment in knee OA patients

Within the OA group, significant negative correlations were observed between the modified KAM and the HKAA or %MA (HKAA: *r* = −0.452; *p* = 0.0302; %MA: *r* = −0.435; *p* = 0.040). In contrast, neither of the KAM impulse nor ΔMME alone showed significant correlation with lower limb alignment parameters (Table 4).

**Table 4:** Correlations between biomechanical variables and radiographic lower‐limb alignment parameters in patients with knee osteoarthritis.

Variable	HKAA	%MA	MTPA	JLCA
Modified KAM	*r* = −0.452*	*r* = −0.435*	*r* = −0.200	*ρ* = 0.290
KAM impulse	*ρ* = −0.370	*ρ* = −0.316	*ρ* = −0.252	*ρ* = −0.072
ΔMME	*r* = −0.186	*r* = −0.200	*r* = −0.012	*ρ* = 0.286

*Note*: Values are presented as Pearson's correlation coefficient (*r*) or Spearman's rank correlation coefficient (*ρ*).

Abbreviations: HKAA, hip‐knee‐ankle angle; JLCA, joint line convergence angle; KAM impulse, impulse of knee adduction moment; Modified KAM, modification between knee adduction moment and ΔMME; MTPA, medial tibial plateau angle; %MA, percentage of mechanical axis; ΔMME, the difference in medial meniscus extrusion between the minimum and maximum.

* Show significant correlation (*p* < 0.05).

## DISCUSSION

This study demonstrated that KAM modified by MME behaviour (ΔMME) effectively distinguished patients with knee OA from healthy individuals, even at the early stages of the disease, and was significantly associated with varus knee alignment. Although ΔMME alone also distinguished patients with knee OA from healthy individuals, it was not significantly associated with any of the radiographic parameters related to varus alignment. These findings support the present hypotheses and suggest that modified KAM may serve as a sensitive biomechanical indicator of mechanopathology in knee OA involving abnormalities in the medial compartment.

The present data showed that both ΔMME and modified KAM were significantly greater in patients with knee OA than in younger and older healthy adults, whereas KAM impulse did not differ significantly between groups. These findings indicate that the MME behaviour contributes to the additional information beyond KAM alone and may represent a more sensitive feature for characterising knee OA. KAM has been widely recognised as a promising surrogate measure for medial compartment loading; however, its interpretation requires caution. Previous studies reported that KAM is influenced by gait speed [39, 43]. Consistent with these observations, the present study demonstrated similar trends in peak KAM and gait speed, with both variables being significantly higher in healthy younger adults than in patients with knee OA. Importantly, KAM itself does not account for individual pathological changes within intraarticular tissues, such as degeneration of the medial meniscus. Knee OA is frequently accompanied by MME, which reflects the impairment of the meniscal hoop function and may alter mechanical loading despite similar external loading conditions [24, 29]. In the present study, the MME was simultaneously measured with the KAM during walking. Therefore, the combined evaluation of MME and KAM may capture functional changes in intraarticular tissues that cannot be sufficiently explained by external loading alone, thereby providing a more comprehensive characterisation of the mechanical pathology associated with knee OA.

Varus knee alignment has a substantial impact on the progression and mechanopathology of knee OA, and its association with biomechanical loading has been consistently demonstrated [7, 38, 44]. In the present study, the modified KAM showed a significant correlation with HKAA or %MA in patients with early‐stage knee OA, whereas neither conventional MME nor MME behaviour exhibited such a relationship. These findings suggest that the modified KAM more consistently reflects the mechanopathological features of early‐stage knee OA and may be a more stable indicator than either MME or KAM alone. KAM represents varus loading acting on the medial compartment during the stance phase of gait and generally increases with disease severity [22]. In contrast, MME behaviour is influenced not only by mechanical loading, but also by meniscal pathology, including tears and their location, even in the early stages of knee OA [2]. Consequently, in early‐stage OA, where varus deformity remains relatively mild, these additional factors may obscure the relationship between MME behaviour and radiographic outcomes. Although the present findings are exploratory and should therefore be interpreted with caution, the modified KAM, which emphasises the influence of mechanical loading while incorporating meniscal dysfunction, may serve as a complementary biomechanical indicator, particularly in the early stages of knee OA when mechanical loading abnormalities are relatively subtle.

BMI and gait speed differed significantly among the three groups. Both variables may influence external knee loading and, therefore, should be considered when interpreting between‐group differences in the modified KAM. BMI is closely related to body mass, which affects ground reaction forces, a determinant of KAM impulse. Thus, BMI is also related to the external mechanical loading component incorporated into the modified KAM, and statistical adjustment for BMI may partially remove variation in mechanical loading that this composite measure is intended to capture. Accordingly, the results of the analyses adjusted for BMI should be interpreted with caution. However, to determine whether the between‐group differences in the modified KAM persisted after accounting for BMI and gait speed, the present study performed additional sensitivity analyses that included these variables as covariates. Generalised linear models demonstrated that the main effect of group on the modified KAM remained significant after adjustment for BMI alone, gait speed alone and both BMI and gait speed simultaneously. Furthermore, pairwise comparisons continued to show a significant difference between the older and OA groups across all models (Table S1). These findings suggest that the difference in the modified KAM between the older and OA groups was not fully explained by differences in BMI or gait speed. Thus, the observed group differences appear to reflect factors other than the magnitude of external loading attributable to body size or walking speed. Given that the modified KAM integrates external knee loading with meniscal deformation, it may provide a composite mechanical measure of interaction between external loading and the mechanical response of the meniscus.

Although emerging serum and imaging biomarkers have improved the detection of early knee OA [27], effective preventive strategies that can be implemented following early diagnosis remain limited. Because knee OA is strongly influenced by mechanical factors, identifying pathological mechanical stress is essential for preventive interventions [13]. Varus knee alignment is represented as the mechanical load on the medial compartment and is a well‐established predictor of knee OA progression [14, 42, 44]. Malalignment is an important target for tibial osteotomy. However, the optimal timing and advantages of alignment‐correcting intervention remain controversial, particularly in the early stages of knee OA [35]. Conservative treatments, including exercise therapy and orthotic interventions, are generally recommended as first‐line management but have limited ability to directly correct structural varus malalignment [3, 4]. Consequently, alignment‐based measures alone may be insufficient for evaluating treatment responses across the spectrum of knee OA management. Previous studies have shown that both KAM and MME can be modified by surgical and nonsurgical interventions [18, 19]; however, the most sensitive biomechanical outcome measure remains unclear. Although ΔMME differed among groups, it was not associated with mechanical factors such as HKAA. In contrast, the modified KAM was associated with these mechanical factors, suggesting that integrating meniscal deformation with external knee loading may better reflect pathological mechanical loading than using either measure alone. Therefore, this integrated metric may be a clinically meaningful outcome measure for monitoring treatment responses and supporting treatment decision‐making in patients with early‐stage knee OA.

This study had some limitations. First, the sample size was relatively small, preventing subgroup analyses based on meniscal characteristics such as tear status and lesion location. Second, radiographic examinations were not performed in healthy individuals, making it difficult to exclude the presence of asymptomatic knee OA. Third, mechanical loading was assessed using noninvasive biomechanical methods, including three‐dimensional motion analysis, rather than direct measurements of intraarticular loading. Therefore, further validation of the observed biomechanical measures using validated musculoskeletal modelling or finite element analysis is warranted. Future studies should incorporate assessments of joint pressure, obtain radiographic evaluations for all participants, and recruit larger cohorts to further validate the clinical utility of the modified KAM as a biomechanical indicator of knee OA mechanopathology.

## CONCLUSIONS

MME during gait moderates the identification of mechanical loading characteristics in the early stages of knee OA and may provide an additional biomechanical indicator to highlight the features associated with mechanical loading stress in the medial compartment.

## AUTHOR CONTRIBUTIONS

**Yosuke Ishii**: Acquisition and analysis of data, conception and design and drafting the article. **Kyohei Nakata**: Recruiting the participants and analysis of data. **Nekomoto Akinori**: Recruiting the participants and analysis of data. **Atsuo Nakamae**: Recruiting the participants. **Masakazu Ishikawa**: Interpretation of data. **Yuko Nakashima**: Obtaining the ethical approval and the established method. **Shigeru Sato**: Acquisition and analysis of data. **Naoto Fujita**: Interpretation of data. **Nobuo Adachi**: final Approval of the manuscript.

## FUNDING INFORMATION

This study was supported with Grants‐in‐Aid for Scientific Research from the Japan Society for the Promotion of Science (JSPS) (Award Numbers: 24K14380).

## CONFLICT OF INTEREST STATEMENT

Yosuke Ishii and Yuko Nakashima declare that they have the lendable device of ultrasound from KONICA MINOLTA, INC. Kyohei Nakata, Akinori Nekomoto, Atsuo Nakamae, Masakazu Ishikawa, Shigeru Sato, Naoto Fujita and Nobuo Adachi that they have no conflict of interest.

## ETHICS STATEMENT

This research has been approved by the authors' affiliated institutions (E2016‐0449). All participants provided informed consent. All participants provided informed consent and signed the agreement.

## Supporting information

Supporting File 1.

## Data Availability

The datasets generated during and/or analysed during the current study are available from the corresponding author on reasonable request.
