# Supplementary material for: Medial meniscus extrusion during gait identifies altered knee mechanical loading in early osteoarthritis of the knee
Source: J Exp Orthop. 2026 Sep 15;13(3):e70910. doi: 10.1002/jeo2.70910 (PMC13577188; doi:10.1002/jeo2.70910)
Supplement: Supplementary file 1 [file JEO2-13-e70910-s001.docx]

**Supplementary Table1:**

Sensitivity analysis of Modified KAM after adjustment for potential covariates

| Model  (Adjustment) | Group effect | Younger / Older | OA / Younger | OA / Older |
| --- | --- | --- | --- | --- |
| 1  (Unadjusted) | χ²(2) = 19.54,  p < .001 | 1.26 (0.79–2.00),  p = .675 | 1.60 (1.07–2.41), p = .018 | 2.02 (1.33–3.06), p < .001 |
| 2  (BMI) | χ²(2) = 12.06, p = .002 | 1.37 (0.86–2.19), p = .297 | 1.31 (0.84–2.04), p = .408 | 1.80 (1.18–2.73), p = .003 |
| 3  (Gait speed) | χ²(2) = 16.56, p < .001 | 1.45 (0.84–2.49), p = .283 | 1.39 (0.82–2.33), p = .380 | 2.01 (1.31–3.08), p < .001 |
| 4  (BMI + gait speed) | χ²(2) = 12.50, p = .002 | 1.57 (0.92–2.68), p = .124 | 1.15 (0.67–1.96), p = 1.000 | 1.80 (1.18–2.76), p = .004 |

Sensitivity analyses were performed to determine whether between-group differences in Modified KAM persisted after adjustment for BMI and gait speed. Because Modified KAM showed a positively skewed distribution, generalized linear models assuming a gamma distribution with a log link function were used, with Modified KAM as the dependent variable and group as the main independent variable. The overall group effect in each model was evaluated using a Type III Wald chi-square test; the χ² statistic and corresponding p value in the “Group effect” column indicate whether Modified KAM differed overall among the three groups after the specified adjustment. Pairwise comparisons are presented as ratios of estimated Modified KAM values with Bonferroni-adjusted 95% confidence intervals and p values. Ratios were calculated in the direction indicated by each column heading (numerator/denominator); thus, a ratio greater than 1 indicates a higher estimated Modified KAM in the numerator group. BMI, body mass index; KAM, knee adduction moment; OA, osteoarthritis.
